# The academic hedge Part I: Modal tuning in your research writing

**DOI:** 10.1007/s40037-019-00559-y

**Published:** 2020-01-17

**Authors:** Lorelei Lingard

**Affiliations:** Schulich School of Medicine & Dentistry, Centre for Education Research & Innovation and Department of Medicine, Health Sciences Addition, University of Western Ontario (Western University), London, Ontario Canada

*The cautious seldom err*.

*~Confucious* [1]

In the Writer’s Craft section we offer simple tips to improve your writing in one of three areas: Energy, Clarity and Persuasiveness. Each entry focuses on a key writing feature or strategy, illustrates how it commonly goes wrong, teaches the grammatical underpinnings necessary to understand it and offers suggestions to wield it effectively. We encourage readers to share comments on or suggestions for this section on Twitter, using the hashtag: #how’syourwriting?

One of the reasons academic conferences are so popular (in addition to geography and climate) is that interacting with other researchers ‘in the flesh’ can be great fun. Especially outside the formal program, in the cafes and bars of the conference city. Over tiny Dutch coffees or goblets of Spanish wine, scholars share the latest twists and turns, successes and sufferings, agreements and arguments in the field. They laugh, they contradict, they argue—mostly with collegial vigour! But have you ever wondered, opening the latest issue of a journal, what’s happened to these dynamic, engaging conversationalists? Why do they seem to be ‘writing without conviction’ [2]?

Researchers can seem quite mincing and modest creatures in their writing. They follow an implicit set of linguistic conventions to represent themselves as cautious, even uncertain as they critique existing knowledge and offer their own knowledge claims. This is the ‘academic hedge’, long regarded as critical to scientific discourse and readily (even semi-consciously) performed by the experienced writer, but difficult to reproduce for the novice or the non-native English writer [3, 4]. When should your writing express confidence, and when should it be tentative? When should you be assertive, and when deferential? Too little hedging, and you can come across as naïve, brash, even rude. Too much, and you can sound as if you don’t have control of the literature, or you don’t believe your own results. The next pair of Writer’s Craft instalments introduce epistemic modality and politeness theory in order to help you to use the academic hedge skilfully.

## Epistemic modality

Writers express their judgment about the degree of probability or factual status of a proposition by using ‘epistemic modality’. In research writing, where ideas—particularly new ones—are only ordained as knowledge by community consensus, a robust set of tools exists to express degrees of probability and factual status. The six sentences in Tab. 1 illustrate this spectrum, from weakest to strongest probability.

**Table 1:** Epistemic modality spectrum

It may be true that medical school structures reproduce social hierarchies	I wonder if it is true that medical school structures reproduce social hierarchies	It is probably true that medical school structures reproduce social hierarchies		I think it is true that medical school structures reproduce social hierarchies	It must be true that medical school structures reproduce social hierarchies		It is certainly true that medical school structures reproduce social hierarchies
Weak/speculative		Intermediary/probabilitive			Strong/assertive		

Epistemic modality is produced through modal auxiliary (helper) verbs (e.g., may, might, must) and adverbs (e.g., possibly, probably, certainly), as well as through lexical (main) verbs that express degrees of certainty (e.g., wonder, think, believe, know).

We could add two more columns to this table. On the far left side, an even more speculative expression would be a question: ‘Is it true that medical school structures reproduce social hierarchies?’ On the far right side, the strongest expression would not be marked by modality at all: ‘Medical school structures reproduce social hierarches.’ When there is no modality present, as in this last example, the writer is making no claim to knowledge; instead, this sentence represents the invocation of common knowledge [5].

Tab. 2 offers a partial list of modal verbs and adverbs that you can use to express degrees of possibility and certainty in your writing.

**Table 2:** Selected verbs and adverbs to express epistemic modality

Certainty	Auxiliary verbs	Lexical verbs	Modal adverbs
Strong	Will, cannot, must	Know, understand, argue, affirm, stress, emphasize, maintain, declare, stipulate, explain, warn, conclude, clarify, identify, insist	Undoubtedly, always, never, definitely, clearly, certainly, obviously, entirely, completely, increasingly
Moderate	Should, would, can, ought to, tends to	Comment, explain, indicate, note, observe, state, describe, identify, find, show, suggest	Usually, likely, probably, regularly, generally, often, frequently, rarely, over the past decade
Weak	May, might, could	Speculate, wonder, believe, note, offer, view, suspect, suggest, consider, propose, debate	Possibly, conceivably, occasionally, tentatively, perhaps, maybe, recently, less, currently, apparently, reportedly

As the above examples illustrate, a key function of epistemic modality is to indicate the degree of certainty of a proposition and the writer’s confidence in it. It offers, therefore, an essential tool when you are reviewing the literature and mapping the gap that your own work will fill. Modality is not a simple recipe, however. It is relational and contextual; therefore, many of the words in Tab. 2 can shift category, as the examples that follow in this piece will illustrate.

The good news about this is that it allows writers to project nuanced degrees of certainty. The bad news is that, if you’re not careful, you can express strong or weak certainty when you don’t intend it. And restricting yourself to verbs and adverbs in the ‘moderate’ category isn’t a solution; this can create limp writing, in which you appear to be simply summarizing knowledge rather than evaluating and controlling it to create an argument. Instead, think carefully about how you are putting together a variety of modal words in your sentences.

Combinations are the key to nuanced author positioning. For instance, you can combine a verb expressing strong certainty with an adverb that weakens it: ‘Researchers have tentatively concluded that’; alternately, you can strengthen a moderate verb with an adverb projecting more certainty: ‘Medical educators increasingly find’. Adverbs of time also have shifting meanings: ‘Recently, a review suggested that’ can project stronger certainty because the knowledge is very current, or weaker certainty because the knowledge has yet to gain the community’s approval, signalled by others taking up the knowledge and reproducing it over time.

Given this complexity, how should the careful writer proceed? Overall, think of ‘tuning’ your certainty up and down, like the volume knob on an old stereo. First, ask yourself whether you have strong, moderate or weak certainty in a knowledge claim you are reviewing or making, and then tune it accordingly. For instance, you can use verbs that signal strong certainty when you are summarizing well-accepted knowledge in your field:

*Clinical teaching requires a balance of patient safety and learner development.*

Expressed with such certainty, this claim appears to be common knowledge; it may not even need a reference. If that seems too certain, then locating or attributing that knowledge is a way to tune the certainty down just a bit. The example below achieves this by locating the knowledge in time and attributing it to a group:

*Over the past decade, researchers have described clinical teaching as requiring a balance of patient safety and learner development.*

You can further tune the certainty up or down by altering the temporal reference. ‘*Recently*, researchers have described …’ would weaken the expression of certainty, while ‘*A generation of* research has described …’ would strengthen it. Here is another example that alters the certainty of the initial claim:

*Sociological research demonstrates that clinical teaching requires a balance of patient safety and learner development.*

In this second example, ‘Sociological research’ is an attribution to a scholarly field that lessens the certainty. Attributions lessen certainty because they retain the flavour of a knowledge *claim*; as Myers points out, any reference to a source lessens the factual status of a claim [5], even when that source is well-regarded. However, in this instance, the claim retains its sense of certainty because of the verb ‘demonstrates’. Choosing another verb, such as ‘suggests’ or ‘proposes’, would tune the certainty down even further, as would citing a person rather than a whole discipline, as in ‘*Carruthers et al.* demonstrate’.

The whole point of doing, and publishing, research is to work in the spaces of moderate and weak certainty: things we do not yet know for sure, expressions of new ideas, debates about alternative conceptualizations. This is why you need to become fluent in weaving together different degrees of modality and, in particular, carefully expressing moderate and weak modality: not because others’ ideas are wrong, but because we are all engaged in a conversation to continuously refine and advance a body of knowledge. Consider how the following passage marks summaries of existing knowledge with modality [6]. I have highlighted verb and adverb constructions that project **strong**, ***moderate ***and *weak *degrees of certainty as the writers state the problem and gap that their work addresses:

To be a student in medical school *may be* stressful^1–3^. Previous studies ***have shown relatively*** high levels of distress, such as symptoms of depression^4,5^ and suicidal thoughts ^6,7^ in medical undergraduates. ***Less is known*** about what conditions encourage positive mental health, and a ***recent*** review of research on medical student distress **emphasised** the need for research concerning the factors that promote well-being^8^. Despite increased attention being paid to positive psychological health and well-being **during the past decades**^9,10^, *only a few* studies have ***focused*** on life satisfaction and coping in medical students. Of these, one study ***found*** that problem focused and emotion focused coping related positively to physical health in first year medical students^11^, and another study ***found*** that coping strategies characterised by engagement predicted fewer symptoms of depression compared to disengagement strategies^12^. A qualitative study of medical student perceptions of an elective wellness course ***reported*** positive responses from the students^13^. A *recent* study **concluded** that personal statements and referees’ reports used in medical school applications **cannot** predict who will be satisfied or dissatisfied with a medical career^14^. ***To date***, **no** longitudinal study ***has identified*** predictors of sustained high levels of life satisfaction among medical students.

First, this passage illustrates how commonly writers combine multiple modals to nuance the degree of certainty in their writing. Second, it shows us that modality is used on two levels: to project the writers’ certainty about knowledge and to represent the certainty of other scholars who authored the knowledge. In the third sentence, the writers project moderate certainty by positioning the review as ‘recent’ and acknowledging that ‘less is known’ about this area, but they assign strong certainty to the authors of the review who ‘emphasized’ the need for more research. Attributing strong certainty to these authors is a rhetorical move that helps make the argument for the writers’ work. This same strategy is visible in the writers’ use of strong modals such as ‘conclude’, ‘cannot’ and ‘no’ in the final two sentences, marking the knowledge gap with confidence.

Finally, the way this passage opens deserves attention, because it raises the question, what is the ‘right’ degree of certainty to project? The opening sentence surprises me with its projection of weak certainty regarding the main problem statement, using the modal auxiliary ‘may be’ and offering references to attribute the knowledge that medical students are stressed. The projection of uncertainty around this central claim makes me as a reader wonder if there is some debate about the existence of stress in medical school. If so, this debate doesn’t manifest in the rest of the paragraph. So perhaps it is just that the authors are performing an academic hedge, starting out with a cautious, uncertain tone as a way of conveying a neutral, ‘scientific’ stance as they open the paper.

This example raises the critical question: when is the academic hedge weakening your position as a writer rather than strengthening it? Getting modal ‘tuning’ right is difficult, both for native English writers and for those writing in English as an additional language (EAL). One pattern of difficulty is that EAL writers may project stronger certainty than they intend, making ‘categorical’ assertions inappropriately [3]. Another pattern of difficulty is that writers new to a scholarly conversation may project artificially weak degrees of certainty, as a way of managing the threat associated with wading into the scholarly fray. The next instalment of this Writer’s Craft will consider this situation in more detail. For now, my message is to be conscious and strategic about how you project certainty in your writing. Ask yourself, what do I think is true? What do my readers think? How can modality help me negotiate the relationship between those positions?

## Conclusion

By wielding epistemic modal verbs and adverbs with skill, you can project degrees of certainty strategically and effectively in your writing. It is not a question of having ‘too much’ or ‘too little’ modality: scientific discourse is always modalized. The question is *how* you have modalized—how you have expressed your judgment about the degree of probability or factual status of the points in your argument—and whether this modal tuning serves your rhetorical purposes.
